# VGF-derived peptide TLQP-21 modulates microglial function through C3aR1 signaling pathways and reduces neuropathology in 5xFAD mice

**DOI:** 10.1186/s13024-020-0357-x

**Published:** 2020-01-10

**Authors:** Farida El Gaamouch, Mickael Audrain, Wei-Jye Lin, Noam Beckmann, Cheng Jiang, Siddharth Hariharan, Peter S. Heeger, Eric E. Schadt, Sam Gandy, Michelle E. Ehrlich, Stephen R. Salton

**Affiliations:** 1 Department of Neurology, Icahn School of Medicine at Mount Sinai, One Gustave L. Levy Place, New York, NY 10029 USA; 2 Guangdong Provincial Key Laboratory of Malignant Tumor Epigenetics and Gene Regulation, Sun Yat-sen Memorial Hospital, Sun Yat-sen University, Guangzhou, Guangdong China; 3 Medical Research Center of Sun Yat-sen Memorial Hospital, Sun Yat-sen University, Guangzhou, Guangdong China; 4 Nash Family Department of Neuroscience, Icahn School of Medicine at Mount Sinai, One Gustave L. Levy Place, New York, NY 10029 USA; 5 Department of Genetics and Genomic Sciences, Icahn School of Medicine at Mount Sinai, One Gustave L. Levy Place, New York, NY 10029 USA; 6 Icahn Institute of Genomics and Multiscale Biology, Icahn School of Medicine at Mount Sinai, One Gustave L. Levy Place, New York, NY 10029 USA; 7 Department of Medicine, Translational Transplant Research Center, Immunology Institute, Icahn School of Medicine at Mount Sinai, New York, NY USA; 8 Sema4, Stamford, CT 06902 USA; 9 Department of Psychiatry and Alzheimer’s Disease Research Center, Icahn School of Medicine at Mount Sinai, New York, NY 10029 USA; 10 Friedman Brain Institute, Icahn School of Medicine at Mount Sinai, One Gustave L. Levy Place, New York, NY 10029 USA; 11 Department of Pediatrics, Icahn School of Medicine at Mount Sinai, New York, NY 10029 USA

**Keywords:** Alzheimer, Microglia, VGF, TLQP-21, C3aR1, Complement

## Abstract

**Background:**

Multiomic studies by several groups in the NIH Accelerating Medicines Partnership for Alzheimer’s Disease (AMP-AD) identified VGF as a major driver of Alzheimer’s disease (AD), also finding that reduced VGF levels correlate with mean amyloid plaque density, Clinical Dementia Rating (CDR) and Braak scores. VGF-derived peptide TLQP-21 activates the complement C3a receptor-1 (C3aR1), predominantly expressed in the brain on microglia. However, it is unclear how mouse or human TLQP-21, which are not identical, modulate microglial function and/or AD progression.

**Methods:**

We performed phagocytic/migration assays and RNA sequencing on BV2 microglial cells and primary microglia isolated from wild-type or C3aR1-null mice following treatment with TLQP-21 or C3a super agonist (C3aSA). Effects of intracerebroventricular TLQP-21 delivery were evaluated in 5xFAD mice, a mouse amyloidosis model of AD. Finally, the human HMC3 microglial cell line was treated with human TLQP-21 to determine whether specific peptide functions are conserved from mouse to human.

**Results:**

We demonstrate that TLQP-21 increases motility and phagocytic capacity in murine BV2 microglial cells, and in primary wild-type but not in C3aR1-null murine microglia, which under basal conditions have impaired phagocytic function compared to wild-type. RNA sequencing of primary microglia revealed overlapping transcriptomic changes induced by treatment with TLQP-21 or C3a super agonist (C3aSA). There were no transcriptomic changes in C3aR1-null or wild-type microglia exposed to the mutant peptide TLQP-R21A, which does not activate C3aR1. Most of the C3aSA- and TLQP-21-induced differentially expressed genes were linked to cell migration and proliferation. Intracerebroventricular TLQP-21 administration for 28 days via implanted osmotic pump resulted in a reduction of amyloid plaques and associated dystrophic neurites and restored expression of subsets of Alzheimer-associated microglial genes. Finally, we found that human TLQP-21 activates human microglia in a fashion similar to activation of murine microglia by mouse TLQP-21.

**Conclusions:**

These data provide molecular and functional evidence suggesting that mouse and human TLQP-21 modulate microglial function, with potential implications for the progression of AD-related neuropathology.

## Background

The *Vgf* (non-acronymic) gene encodes a neuronal and neuroendocrine protein precursor [1] that is post-translationally processed with cell- and tissue-type specificity into multiple bioactive peptides that are secreted and are involved in numerous physio/pathological functions including reproduction [2], depression [3, 4], obesity [5], memory [6] and also neurodegenerative diseases, in particular Alzheimer’s disease (AD). In AD patients, VGF-derived peptides are reduced in the cerebrospinal fluid (CSF), suggesting their potential utility as biomarkers and a possible role for VGF in AD pathogenesis and progression [7–14]. Recent studies conducted by the NIH Accelerating Medicines Partnership for Alzheimer’s Disease (AMP-AD) consortium have further identified reduced VGF levels in the brains of AD subjects that correlate with mean amyloid plaque density, Clinical Dementia Rating (CDR) and Braak scores, with multi-omic network analysis further indicating that VGF is a key driver of AD pathogenesis and progression [15, 16]. The C-terminal peptide TLQP-21 (named by its four N-terminal amino acids and length) is processed from the 617 amino acid VGF precursor, is expressed in the brain [17], and plays a role in the central and peripheral nervous system (CNS and PNS) to regulate feeding, reproductive and circadian behaviors, body weight, neuropathic pain and peripheral adiposity [17–23]. The C3a receptor-1 (C3aR1), a 7-transmembrane spanning G-protein coupled receptor that is activated by the complement activation product C3a (traditionally considered a component of innate immunity), was identified as a target for TLQP-21 [24, 25]. C3aR1 is expressed by neurons, astrocytes, and microglia in the brain [26–28], but the functional consequences of TLQP-21 activation of C3aR1 on microglia are poorly understood [29].

AD is the most frequent form of dementia and no effective treatment is currently available. Glial phagocytosis has been investigated as an underlying mechanism for several neurodegenerative diseases including AD. Phagocytic dysfunction of glial cells can result in the accumulation of amyloid-β (Aβ) in the brain accompanied by an abnormal immune response [30]. Characterized pathologically by the accumulation of Aβ-plaques and neurofibrillary tangles, AD is associated with synaptic failure, dendritic atrophy and inflammation. Microglia, as the innate immune cells of the brain, are strongly involved in these processes, releasing pro-inflammatory cytokines and mediating synaptic pruning via a complement-dependent mechanism [31]. In addition, a majority of the common AD variants identified by GWAS are preferentially expressed in microglia compared to neurons or astrocytes [32, 33], consistent with a substantial role for microglia in AD progression. However, the associated cellular and molecular pathways are not entirely understood.

Here, we examined the effects of TLQP-21 treatment on microglial function using three different in vitro models: the murine BV2 microglial cell line, primary microglia from wild-type (WT) or *C3aR1*-null, i.e. knock-out (KO), mice and the human HMC3 microglial cell line. Using motility and phagocytosis assays as well as RNA sequencing, we identified critical roles for C3aR1 in microglial regulation by TLQP-21 and for the TLQP-21/C3aR1 pathway in microglial migration and phagocytosis. Furthermore, we demonstrated that chronic intracerebroventricular administration of TLQP-21 to 3-month-old 5xFAD mice, a transgenic mouse model of amyloidosis that overexpresses mutant presenilin and amyloid precursor protein [34], results in reduced amyloid pathology and microgliosis. Finally, we demonstrated that the human TLQP-21 peptide similarly activates human microglia, paving the way for further translational studies.

## Methods

### Animals

Breeding pairs of wild-type (WT) C57BL/6 J mice were obtained from the Jackson Laboratories. C3aR1 knock-out (KO) mice on a C57BL/6 J background were backcrossed > 14 generations at Mount Sinai [35]. WT and KO animals were bred in the same room at Mount Sinai to limit any potential effects of microbiomes. For in vivo experiments, TLQP-21 (2.5 mg/ml) dissolved in aCSF or aCSF alone was delivered intracerebroventricularly (icv) by micro-osmotic pump (Alzet delivering 0.25 μl/h or 15 μg/day) at 3 months of age for 28 days. The cannula was implanted at the following coordinates: AP = − 0.1, ML = ±1.0 and DV = − 3.0 from bregma (mm). Male and female mice at 4–5 months of age were anesthetized and perfused for immunohistochemical analysis as described below. Generation of 5xFAD mice has been previously described [34]. These mice overexpress both human APP (695) harboring the Swedish (K670 N, M671 L), Florida (I716V) and London (V717I) familial AD (FAD) mutations and human Presenilin1 (PS1) harboring the two FAD mutations M146 L and L286 V. Expression of both transgenes is regulated by neuronal-specific elements of the mouse Thy1 promoter. The 5xFAD strain (B6/SJL mixed genetic background) was maintained by crossing hemizygous transgenic mice with B6/SJL F1 breeders.

### Tissue collection and sample preparation

Mice were anesthetized in a CO_2_ chamber and transcardially perfused with 20 ml of ice-cold PBS. Brains were post-fixed 16 h in 4% PFA and sliced into 40 μm sections using a vibratome (Leica) for histological analyses. Sections were washed with 0.1% Triton X-100 in PBS, saturated by incubation with 0.1% Triton X-100 in PBS/5% goat serum, and then incubated with primary antibodies as following: 6E10 (1/1000, mouse, Covance), IBA1 (1/1000, rabbit, Wako) and LAMP1 (1/1000, rabbit, Abcam). For non-fluorescent immunostaining, endogenous peroxidase was quenched with PBS containing 3% H_2_O_2_ for 15 min followed by amplification using the ABC system (VECTASTAIN Elite ABC HRP Kit, Vector Laboratories, Burlingame, CA, USA). Horseradish peroxidase conjugates and 3,3′-diaminobenzidine were used according to the manufacturer’s manual (Vector® DAB, Vector Laboratories, Burlingame, CA, USA). Images were obtained with an Olympus BX61 microscope and Zeiss LSM 780. Regions of interest were randomly selected in each brain area to be analyzed (cortex, CA1, DG + Hilus, CA3). The parameters for image acquisition were set at the same level for each immunostained protein. Staining was analyzed by ImageJ software at the same threshold setting for each immunostained marker, among all brain slices examined. For the data analysis of mouse experiments, images were numbered by the person who acquired them, and then another examiner blinded to genotype and treatment analyzed the images.

Samples used for western-blot were homogenized in a RIPA buffer (Pierce) containing phosphatase (Pierce) and protease (Roche) inhibitors, centrifuged for 20 min at 15,000 g and the supernatant was used for the immunoblotting analysis. Membranes were incubated with either anti-VGF C-terminal (1:1000; rabbit polyclonal) [6, 36], anti-actin (1:3000, mouse, Millipore), anti-6e10 (1:1000, mouse, Covance), anti-PSD-95 (1:1000, mouse, Millipore), anti-GAPDH (1:1000, mouse, Santa-Cruz) and anti-tubulin (11,000, mouse, Sigma) antibodies. Optical density was measured and quantified using Fiji software (ImageJ).

### Cell culture

The immortalized murine microglial cell line (BV2) was generated by infecting primary microglial cell cultures with a v-raf/v-myc oncogene carrying retrovirus (J2) [37]. Cells were grown in high glucose DMEM supplemented with 10% heat-inactivated FBS (Gibco), 2 mM glutamine and penicillin/streptomycin (100 U/ml and 0.1 mg/ml respectively) and maintained at 37 °C and 5% CO_2_.

**Fig. 1:**
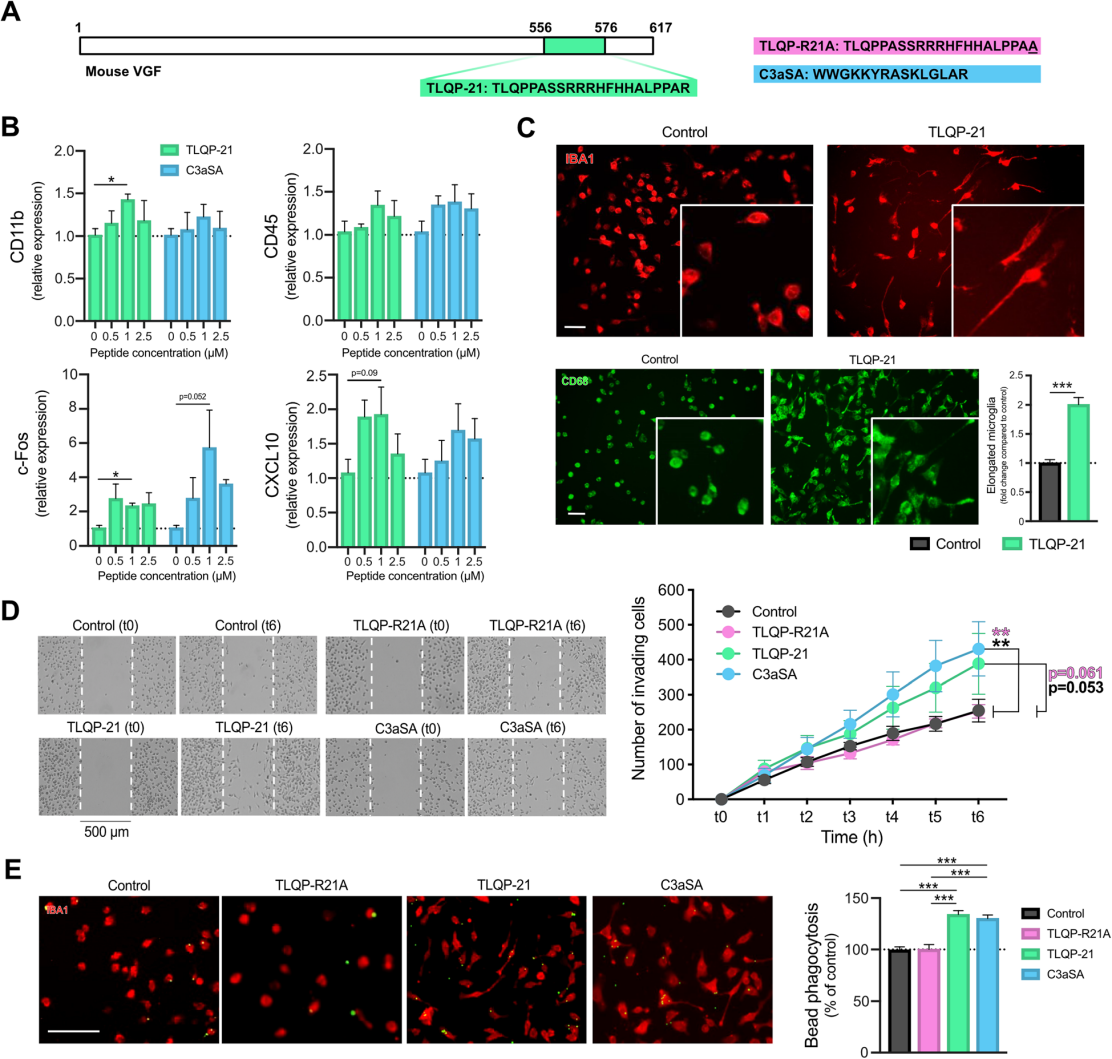
TLQP-21 alters morphology, motility and phagocytosis of BV2 microglia. **a**, Schematic representation of the mouse VGF protein sequence showing TLQP-21 peptide (green), the inactivating amino acid substitution in the mutant TLQP-R21A (pink) and the C3 super-agonist C3aSA (blue). **b**, RT-qPCR quantification of *CD11b*, *CD45*, *c-Fos* and *CXCL10* mRNA levels after 1 h of treatment with TLQP-21 or C3aSA (0 to 2.5 μM). *n* = 2–6 wells/group from one single culture experiment. A Kruskal-Wallis test followed by a Dunn’s multiple comparisons test was used. **c**, Representative images of BV2 cells immuno-labelled with antibodies directed against IBA1 (red) or CD68 (green), following treatment with TLQP-21 (1 μM). The histogram on the bottom-right represents a quantification of the fold-change of elongated BV2 cells following TLQP-21 treatment in comparison to control. *n* = 5–6 wells/group (with 3 photomicrographs per well and an average of 115 cells examined per photomicrograph) with *N* = 3 independent experiments (data presented are the average of all experiments). Scale bar = 50 μm. **d**, Representative photomicrographs of the wound healing assay performed on BV2 cells treated with TLQP-21, TLQP-R21A or C3aSA for 1 h before the scratch. A picture of the same area was taken for each well every hour for 6 h. The graphic on the right represents the number of invading cells in the scratch for each group and time. *n* = 5–6/wells/group (with 1 photomicrograph per well at the exact same position) and *N* = 3 independent experiments (data presented are the average of all experiments), a Two-Way ANOVA test followed by a Tukey’s multiple comparisons test was done. **e**, Representative images of the phagocytosis assay (in red: IBA1 immunostained BV2 cells; in green: fluorescent latex beads) performed on BV2 cells treated with TLQP-21, TLQP-R21A or C3aSA. The graphic on the right represents the percentage of phagocytosed latex beads compared to control. *n* = 18–24 images/group (from 3 to 4 wells/group) with *N* = 3 independent experiments (data presented are the average of all experiments), a One-Way ANOVA followed by a Tukey’s multiple comparisons test was done. Error bars represent means ± SEM. *p < 0.05, ***p < 0.001

Murine primary microglia were isolated from cerebral cortices, dissected from postnatal day P0-P3 wild type (WT) C57BL/6 J and homozygous C3aR1 KO mice (C57BL/6 J background). Briefly, tissue was homogenized in ice-cold PBS then centrifuged at 300 g for 5 min. The pellet was resuspended in the same medium previously described and cells seeded in poly-L-lysine T75 pre-coated flasks. Cultures were maintained at 37 °C and 5% CO_2_ for 2 weeks, during which media change was performed twice a week. After 2 weeks, cultures were agitated at 180 rpm for 30 min to detach microglial cells from the astrocytic monolayer for collection. Before each experiment, cells were serum deprived for 30 min and then were treated with the different peptides for 1 h in serum-free DMEM. Except for Fig. 1b, 1 μM final concentrations of each peptide (TLQP-21, TLQP-R21A or C3aSA) were used. Mouse/rat peptides were purchased from BACHEM and were suspended in 1xPBS at 500 μM and stored as stock solutions at − 80 °C prior to final dilution in DMEM. HMC3 microglia were purchased from ATCC® (CRL-3304, batch number:70016372).

### Wound-healing assay

Immortalized murine microglial BV2 cells were first seeded in 24-well plates at a density of 150,000 cells per well and the wound healing assay was performed 24 h later. A small area was disrupted by applying a scratch on the cell monolayer using a plastic pipette tip and then the medium was changed. Photomicrographs of identical areas in each well were taken every hour for 6 h. The number of invading cells was counted every hour in the same area for each well.

### Phagocytosis assays

Green fluorescent latex beads (Sigma #L1030) and fluorescent 488-labelled human Aβ42 (Anaspec #AS-60479-01) were used to assess phagocytosis in both BV2 cells and primary microglia. Beads were pre-opsonized in heat inactivated FBS (Gibco) for 1 h at 37 °C before use [38]. The final concentrations for beads and FBS in DMEM were 0.01% (v/v) and 0.05% (v/v) respectively. Fluorescent Aβ_42_ was solubilized in a 1% NH_4_OH and used at 5 μM concentration. Briefly, cells were submitted to serum deprivation for 30 min prior to addition of each peptide (1 μM for 30 min), and then fluorescent latex beads or Aβ_42_ were added to the media for an additional 30 min. Cultures were then washed 3 times with PBS and fixed in 4% paraformaldehyde (PFA). DAPI staining was used to count the total number of cells and each IBA1 immunolabeled-cell containing at least one bead or one aggregate of Aβ was counted as positive for phagocytosis. Photomicrographs of randomly selected fields were collected on an inverted microscope at 20x magnification. Bead number was quantified using the “Cell Counter” plugin of Fiji software. For fluorescent Aβ_42_, pictures were thresholded and the percentage of Aβ positive area was calculated using Fiji software and the “measure” function.

### Immunocytochemistry

Cells were washed in PBS three times before fixation for 10 min with 4% paraformaldehyde (PFA). Cells were then incubated with 0.25% TritonX-100 in PBS and blocked with 0.25% Triton X-100 and 1% BSA in PBS. Immunocytochemistry was performed using primary anti-IBA1 (Rabbit, 1:1000; Wako, Richmond, VA) or anti-CD68 (Rat, 1:200, mca1957, AbD Serotec BioRad) antibodies followed by secondary Alexa fluorescent anti-rabbit or anti-rat antibodies (12,000; Invitrogen, Carlsbad, CA).

### RNA extraction and qPCR analysis

RNAs were isolated using the QIAzol® Lysis Reagent (Qiagen) and the miRNeasy® Micro Kit (Qiagen). For RT-qPCR analyses, the abundance of each transcript was normalized to the abundance of L32 with the ΔCt method. The All-in-One qPCR Mix (GeneCopoeia) was used to perform RT-qPCR. The sequences of oligonucleotides used were as follows:

- Cd11b: 5′-AAACCACAGTCCCGCAGAGA-3′ and 5′-CGTGTTCACCAGCTGGCTTA-3′.
- Cd45: 5′-GAACATGCTGCCAATGGTTCT-3′ and 5′-TGTCCCACATGACTCCTTTCC-3′.
- c-Fos: 5′-CCGAAGGGAACGGAATAAGA-3′ and 5′-TGCAACGCAGACTTCT**C**ATCT-3′.
- Cxcl10: 5′-CCAAGTGCTGCCGTCATTTTC-3′ and 5′-GGCTCGCAGGGATGATTTCAA-3′.
- hTrim47: 5′-GAGAGTACGAACCTCTTGGAGA-3′ and 5′-CACCCTCTTGACACCTTTGGT-3′.
- hDusp18: 5′-GCCTTCCCAGTTCAGTTCCG-3′ and 5′-GTGATCTGGTTGCTAGACAGC-3′.
- hLmna: 5′-AATGATCGCTTGGCGGTCTAC-3′ and 5′-CACCTCTTCAGACTCGGTGAT-3′.
- hFurin: 5′-TCGGGGACTATTACCACTTCTG-3′ and 5′-CCAGCCACTGTACTTGAGGC-3′.
- hArl13b: 5′-AAAGAGCTGAACGAGTGCGAA-3′ and 5′-AGACCACTGGTTCCATCGAGT-3′.
- hMtmr10: 5′-GCAAATTGTCACAGTAAACGACC-3′ and 5′-TGGCTGGGAATAATGAGCTATTG-3′.

Primers used in Fig. 6h are previously published [29]. Primers for the human c-Fos used in Fig. 7a were TaqMan® probes (Hs04194186 and Hs02786624 for c-Fos and Gapdh respectively).

### RNA-sequencing

RNA sequencing was performed by Novogene (https://en.novogene.com) using Illumina Novaseq 6000 S4 flow cells. Only samples with RNA integrity number (RIN) > 9 were used. Non-directional libraries were constructed with a NEB kit using the manufacturer’s protocol. RNA sequencing assays were performed after ribosomal RNA depletion by Ribo-Zero. For the data QC, four main steps were implemented including determination of the (1) distribution of sequencing quality, (2) distribution of sequencing error rate, (3) distribution of A/T/G/C bases, and (4) results of raw data filtering. The filtering process included: (1) removal of reads containing adapters, (2) removal of reads containing *N* > 10% (N represents bases that cannot be determined), and (3) removal of reads containing low quality bases (Qscore ≤5) that are over 50% of the total bases contained in the read.

### Analysis of RNA-sequencing data

Genes with at least 1 count per million in 2 or more samples were included in the analyses. Raw count data were normalized by the voom function in the R limma package [39–41]. Principal component analyses were used to define outliers from the gene expression data, and 3 outliers that were more than 2 standard deviations from the centroid were removed. Analyses to define genes that were differentially expressed between conditions (groups) of interest and for each treatment and genotype, were carried out with the lmFit function from the limma R package [39–41]. Differentially expressed genes (DEGs) were defined as having an adjusted *p*-value < 0.05 (Benjamini-Hochberg procedure) and at least 1.2-fold change in expression. For the co-expression network analysis, two co-expression networks were built on the voom normalized counts, one for the WT groups only (taking into account all WT comparisons with the different treatments) and one for all groups (all WT and KO comparisons were used). To construct the coexpression networks, we used the coexpp R package (https://bitbucket.org/multiscale/coexpp). To identify modules of interest, we projected the union of all DEGs on the corresponding co-expression network (adjusted *p* value threshold at 0.1). We calculated enrichment statistics using Fisher’s Exact Test, and corrected for multi-testing using the Bonferroni correction. Module annotation was performed using GO term enrichment using the R packages goseq [42], topGO (Alexa and Rahnenführer 2018), and org. Mm.eg.db (Marc Carlson 2018). Revigo was used to visualize and summarize the GO terms [43]. Ingenuity Pathway Analysis (IPA) software (Qiagen) was used to identify canonical pathways.

### Y-maze test

The Y-maze test is commonly used to assess hippocampal-dependent spatial working memory in rodents [34, 44, 45], with improved memory being directly proportional to increased spontaneous Y-maze alternations (i.e. tendency to enter a less recently visited arm). Mice were placed at the center of the maze and were allowed to explore freely for 5 min. The total number of arms entered and the entry sequence were recorded. The maze was thoroughly cleaned with 70% alcohol after completion of each test. A triad is defined as a set of 3 consecutive arm entries, and an alternation is defined as a triad that consists of 3 unique arm entries (e.g. ABC, BCA or CAB versus ACA or BAB). Percent alternation is calculated as the [number of alternations divided by the total possible alternations] × 100, or [number of alternations/(total entries - 2)] × 100. Chance performance in this task is 50%. Performance of the three groups (WT + aCSF, 5xFAD + aCSF, and 5xFAD + TLQP-21) was analyzed by one-way ANOVA, and any trends obtained by ANOVA were further examined by paired comparison of the respective groups by Student’s t-test.

### Statistics

**Fig. 2:**
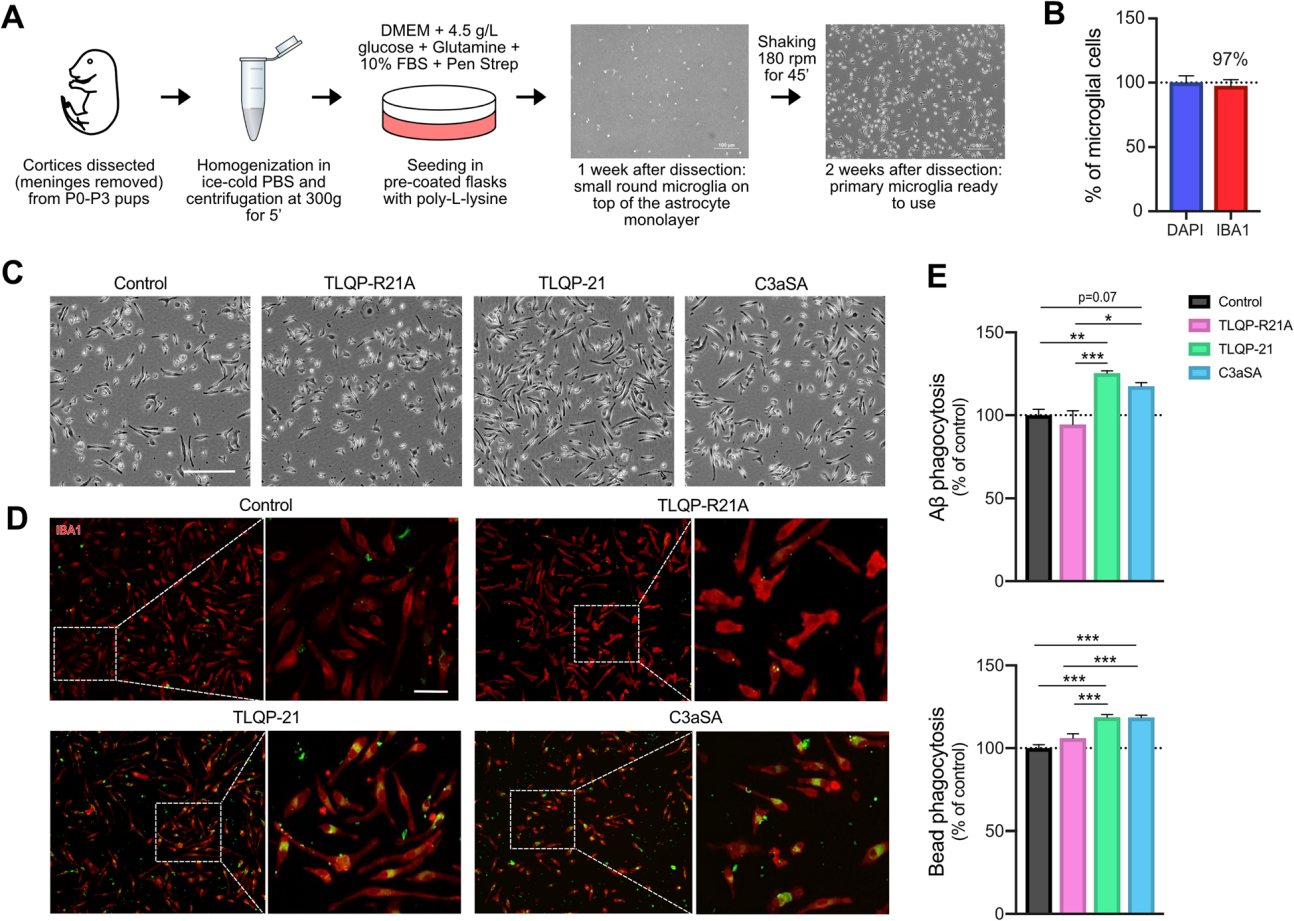
TLQP-21 induces morphological changes and increases Aβ phagocytosis in primary microglia. **a**, Flowchart shows streamlined isolation of primary microglia from C57BL/6 J P0-P3 pups. **b**, Percentage of IBA1 positive cells in primary microglial cultures (an expression of purity). **c**, Representative gray-level images of primary microglia following the different treatments (1 μM TLQP-21, TLQP-R21A or C3aSA for 1 h). Scale bar = 200 μm. **d**, Representative images of human fluorescent synthetic Aβ (Anaspec, green) phagocytosed by primary microglia (red) after treatment with peptide. Scale bar = 50 μm. **e**, Top panel shows the quantification of microglial cells with at least one Aβ aggregate in their cell body (expressed as % control); *n* = 3 photomicrographs/well, 6 wells/group, and *N* = 2 independent experiments (data presented are the average of both experiments). Bottom panel shows the quantification of microglial cells with at least one latex bead in their cell body (expressed as % control); *n* = 4–7 photomicrographs/well, 4–5 wells/group, and *N* = 2 independent experiments (data presented are the average of both experiments). Error bars represent means ± SEM. Statistical analyses were performed using a One-Way ANOVA followed by a Tukey’s post-hoc test, *p < 0.05, **p < 0.01, ***p < 0.001

**Fig. 3:**
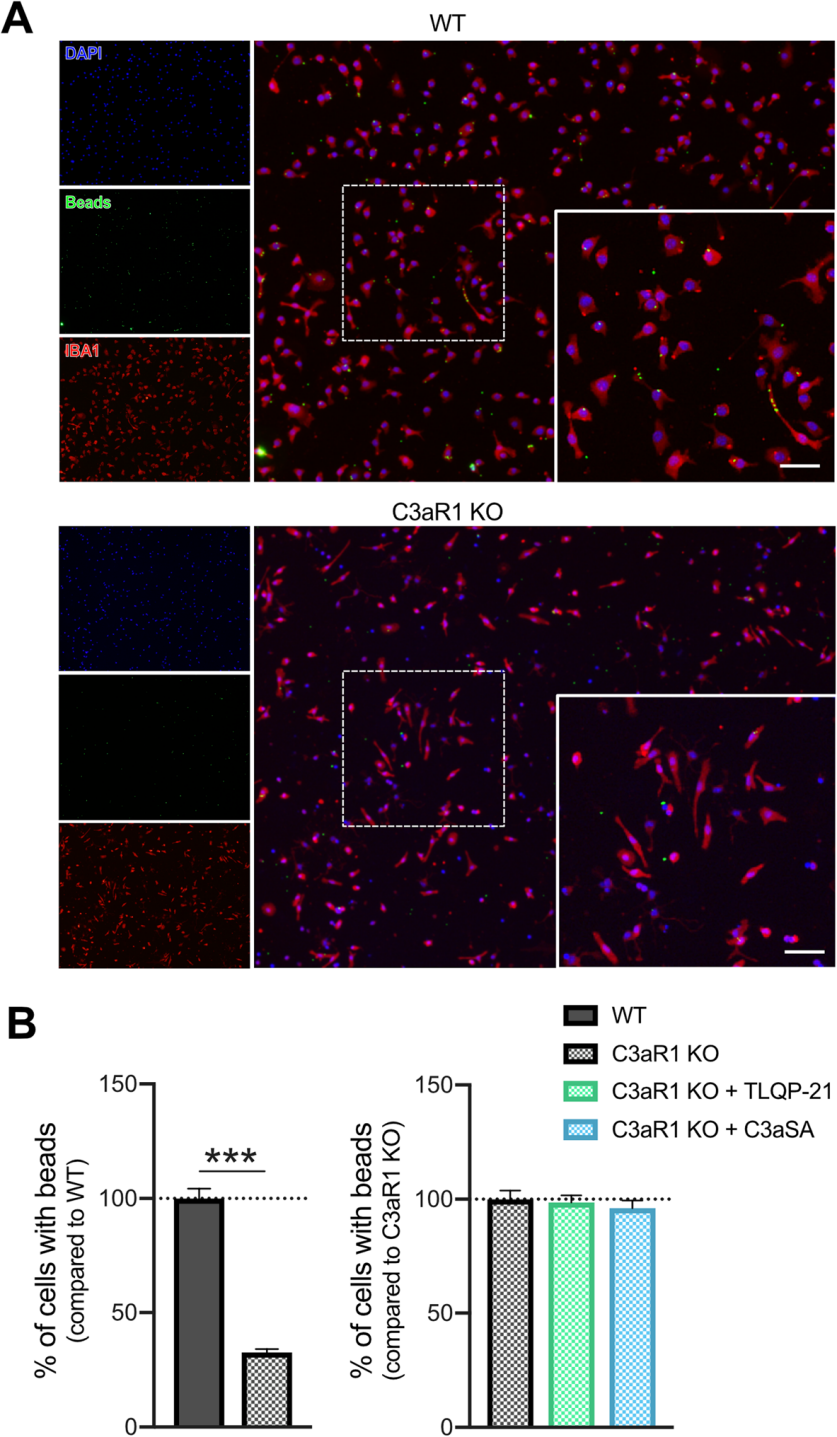
C3aR1 knock-out impairs phagocytosis in primary microglia. **a**, Representative images of latex bead phagocytosis assay on WT primary microglia (top) or C3aR1 knockout (KO) primary microglia (bottom, immunolabelled with IBA1 antibody in red and DAPI in blue). Scale bar = 50 μm. **b**, Left panel: Quantification of C3aR1 KO microglial cells showing at least one latex bead in their cell body (percentage of WT) in the non-treated condition; Right panel: quantification of C3aR1 KO microglial cells showing at least one latex bead in their cell body (% control) after treatment with the different peptides (1 μM TLQP-21 or C3aSA, for 1 h). *n* = 18 photomicrographs/group from *n* = 4–5 wells with *N* = 2 independent experiments (data presented are the average of both experiments). Error bars represent means ± SEM. Statistical analyses were performed using a Student t-test (left histogram) or a One-Way ANOVA followed by a Tukey’s post-hoc test (right histogram), ***p < 0.001

The non-genomic data (Figs. 1, 2, 3, 6 and 7) were analyzed with GraphPad Prism 8. Graphs represent the mean of all samples in each group ± SEM. Sample sizes (n values) and statistical tests are indicated in the figure legends. A one-way ANOVA followed by a Tukey’s post-hoc test was used for multiple comparisons. A Student’s t-test was used for simple comparisons. Significance is reported at *p < 0.05, **p < 0.01, ***p < 0.001.

## Results

### TLQP-21 increases BV2 cell migration and phagocytotic activity

TLQP-21 is a bioactive C-terminal VGF-derived neuropeptide and the R21A mutation of its C-terminal amino acid eliminates its activation of the complement component 3a (C3a) receptor-1 (C3aR1) [25] (Fig. 1a). As C3aR1 is a target of TLQP-21 [5, 24, 25], we compared TLQP-21 with the C-terminal synthetic analogue of C3a (C3aSA) which is derived from the fifteen C-terminal residues (6–77: WWGKKYRASKLGLAR) of the natural C3aR1 agonist C3a and acts as a super-agonist to the C3a receptor [46]. We sought to investigate whether both peptides share intracellular pathways that impact microglial actions and phenotype. Dose-response analyses were carried out to determine optimal concentrations of each peptide. BV2 cells, treated with TLQP-21 or C3aSA (0.5–2.5 μM) for 1 h, were extracted and total RNA analyzed by RT-qPCR for known microglia/macrophage marker mRNAs (Cd11b, Cd45, Cxcl10) and for the immediate early gene *c-Fos*. A dose-dependent increased trend in expression of CD11b, CD45, c-Fos and CXCL10, plateauing after 1 μM peptide treatment, was observed (Fig. 1b). We therefore used 1 μM concentrations, as previously used by others [47], for both peptides to characterize the effects of TLQP-21.

We stained microglia with antibodies to IBA1 and CD68 proteins, which are commonly used to identify microglia and to assess their activation [48]. Based on microscopic examination, there appeared to be a shift in many microglia from round to rod-shaped/elongated morphology after TLQP-21 treatment (Fig. 1c). Next, we assessed the impact of TLQP-21 on cell motility by performing a scratch wound healing assay following exposure to TLQP-21, TLQP-R21A, C3aSA, or vehicle. There were a greater number of invading cells in wells containing TLQP-21 or C3aSA compared to TLQP-R21A or untreated wells (Fig. 1d). Finally, phagocytic function was quantified using a fluorescent latex bead uptake assay. Following the same treatment as described above, we observed 34 and 30% increases in bead phagocytosis following TLQP-21 and C3aSA treatment, respectively (Fig. 1e). No changes were found in the number of cells in TLQP-21 or C3aSA-treated cultures compared to controls over the time course of these experiments (data not shown).

### TLQP-21 via C3aR1 increases bead and Aβ phagocytosis in primary microglia

To confirm and extend our observations with the BV2 cell line, we used murine C57BL/6 J primary microglia as a second model (Fig. 2a). We confirmed that the cell cultures contained 97% microglia by comparing the number of cells stained by IBA1 to the total number of DAPI positive cells (Fig. 2b). Consistent with what was observed in BV2, TLQP-21 exposure increased numbers of elongated microglia compared to untreated cultures (i.e. control or TLQP-R21A; Fig. 2c). We also compared the effects of TLQP-21, TLQP-R21A, and C3aSA on Aβ_42_ uptake in primary microglia. Using a fluorescent human synthetic Aβ_42_, there was a significant increase in microglial phagocytosis in cells treated with TLQP-21 or C3aSA relative to either control or TLQP-R21A treatments (Fig. 2d-e). Phagocytosis assays using fluorescent latex beads showed similar results (Fig. 2e). These data show that TLQP-21 increases microglial migration and phagocytosis.

To determine whether expression of C3aR1 is required for TLQP-21 regulation of microglial phagocytosis, microglia from congenic, homozygous C3aR1 knockout mice were isolated. There was a 63% decrease in basal phagocytosis in C3aR1 KO microglia compared to WT (Fig. 3a-b). Additionally, TLQP-21, TLQP-R21A, and C3aSA all failed to increase phagocytosis in C3aR1 KO microglia (Fig. 3b).

### TLQP-21 induces expression of genes associated with cellular movement and migration

**Fig. 4:**
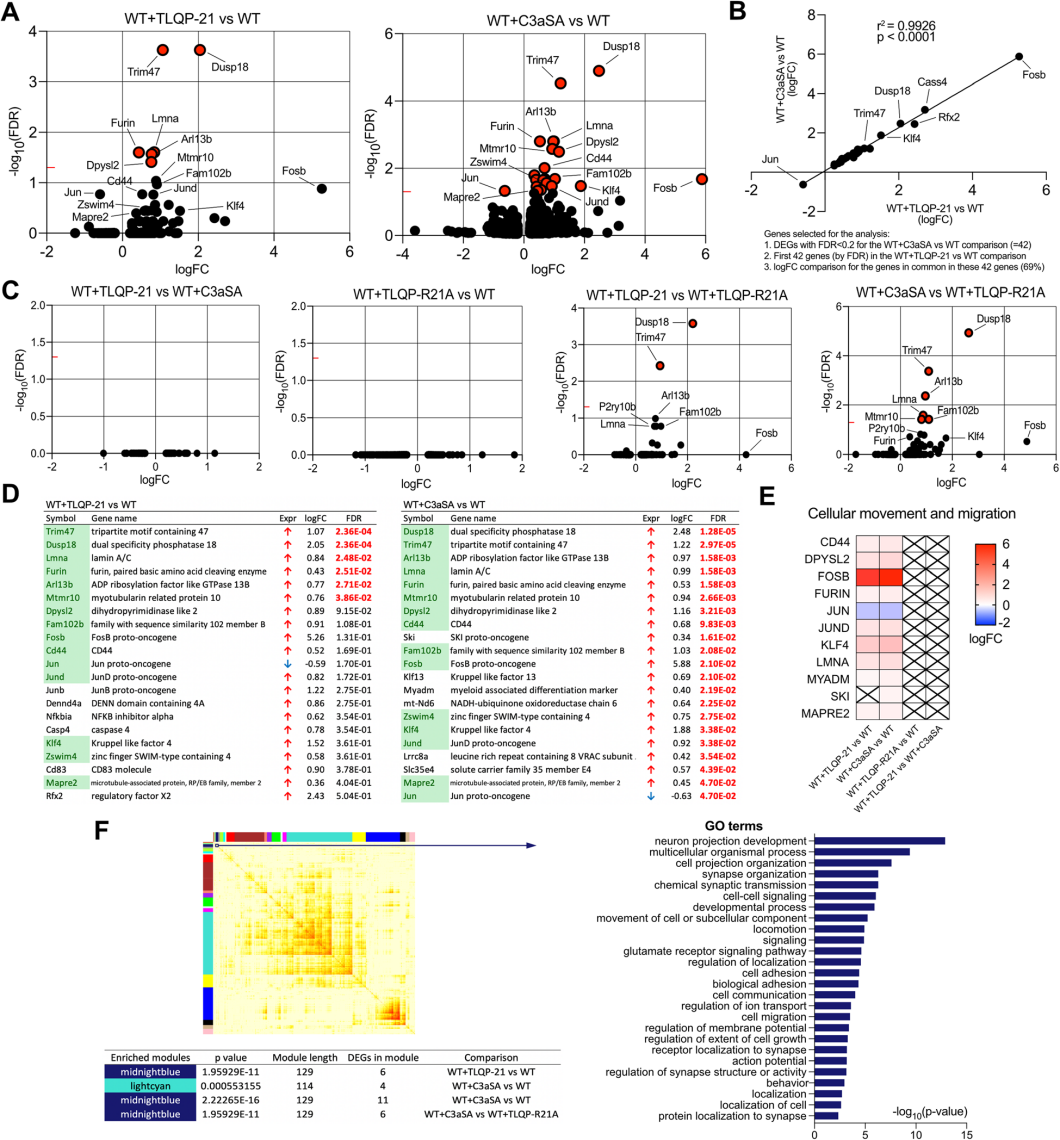
TLQP-21 induced differentially expressed genes (DEGs) are related to cell migration and proliferation. **a**, Volcano plot representations of the DEGs in WT primary microglia treated with TLQP-21 or C3aSA. Red dots represent DEGs at an FDR < 0.05. **b**, Correlation of the fold changes (FC) of DEGs between WT + TLQP-21 vs WT and WT + C3aSA vs WT comparisons. **c**, Additional volcano plot representations of the DEGs in primary microglia treated with TLQP-21, C3aSA or TLQP-R21A. **d**, Tables listing the DEGs (FDR < 0.05) for the WT + C3aSA vs WT comparison (*n* = 21 DEGs) compared to the first most significant 21 genes in the WT + TLQP-21 vs WT comparison (genes shaded green are similarly regulated in the top 21 by TLQP and by C3aSA (~ 70%). Indicated in bold and red are the significant DEGs with FDR < 0.05. Indicated in green are the genes found in both comparisons (≈70% similarity). **e**, Heatmap of significantly affected genes in the most significant canonical pathway found in the WT + C3aSA vs WT comparison using Ingenuity Pathway Analysis (IPA) software (“Cellular movement and proliferation” z-score = 1.091, *p*-value = 8.05E-05). **f**, Network modules identified by weighted gene co-expression network analysis (WGCNA). The co-expression network (13 samples in total) was constructed using all WT-related comparisons. Each row and column correspond to a gene. The modules are indicated by the colored bars next to the heat map. Light color in the heat map indicates low topological overlap and progressively darker red represents higher topological overlap. The right histogram represents GO terms after Gene Ontology (GO) analysis. Each significant GO term was grouped according to its parental ontology to underline highly represented functions using REVIGO

To identify molecular changes induced by TLQP-21 treatment, we generated transcriptomic profiles from WT primary microglia treated with each peptide (TLQP-21, TLQP-R21A, or C3aSA). There were 6 differentially expressed genes (DEGs) in WT microglia treated with TLQP-21 as compared to the untreated WT microglia (WT + TLQP-21 vs WT) and 21 DEGs in WT treated with C3aSA as compared to untreated WT (WT + C3aSA vs WT; Fig. 4a) at an FDR < 0.05. There was a strong correlation of the log fold change responses between these comparisons, suggesting a similar mechanism for both TLQP-21 and C3aSA transcriptional regulation (r^2^ = 0.9926, *p* < 0.0001; Fig. 4b). The 6 DEGs in WT vs TLQP-21 were *Trim 47*, *Dusp18*, *Lmna*, *Furin*, Arl13b, and *Mtmr10*, and these were the top 6 genes in WT vs. C3aSA. Moreover, there were no significant DEGs (FDR < 0.05) when comparing WT + TLQP-21 vs WT + C3aSA (Fig. 4c). As predicted, TLQP-R21A did not induce transcriptional changes relative to WT. We observed a similar distribution in the volcano plots of the DEGs when the WT + TLQP-21 or WT + C3aSA groups were compared to WT or WT + TLQP-R21A (Fig. 4c). Other than *Jun*, all DEGs were upregulated in the comparisons of WT + TLQP21 and WT + C3aSA vs WT and there was a ~ 70% overlap of the top 21 genes between WT + TLQP-21 vs WT and WT + C3aSA vs WT (Fig. 4d). Ingenuity Pathway Analysis (IPA) identified “Cellular movement and proliferation” (z-score = 1.091, *p* value = 8.05E-05) as the most highly affected molecular and cellular function, containing 11 genes (Fig. 4e). Weighted Gene Co-expression Network Analysis (WGCNA) was used to identify modules that correspond to clusters of genes sharing similar biological processes or related functions. Most of the DEGs were detected in the “midnight blue” module, and Gene Ontology (GO) terms associated with this module include several pathways revolving around neuronal projections and synapses and cell-signaling/adhesion/migration (Fig. 4f). No changes in cell cycle related genes or canonical pathways related to cell cycle and chromosomal replication were detected.

### C3aR1 knock-out abolishes TLQP-21 induced DEGs

**Fig. 5:**
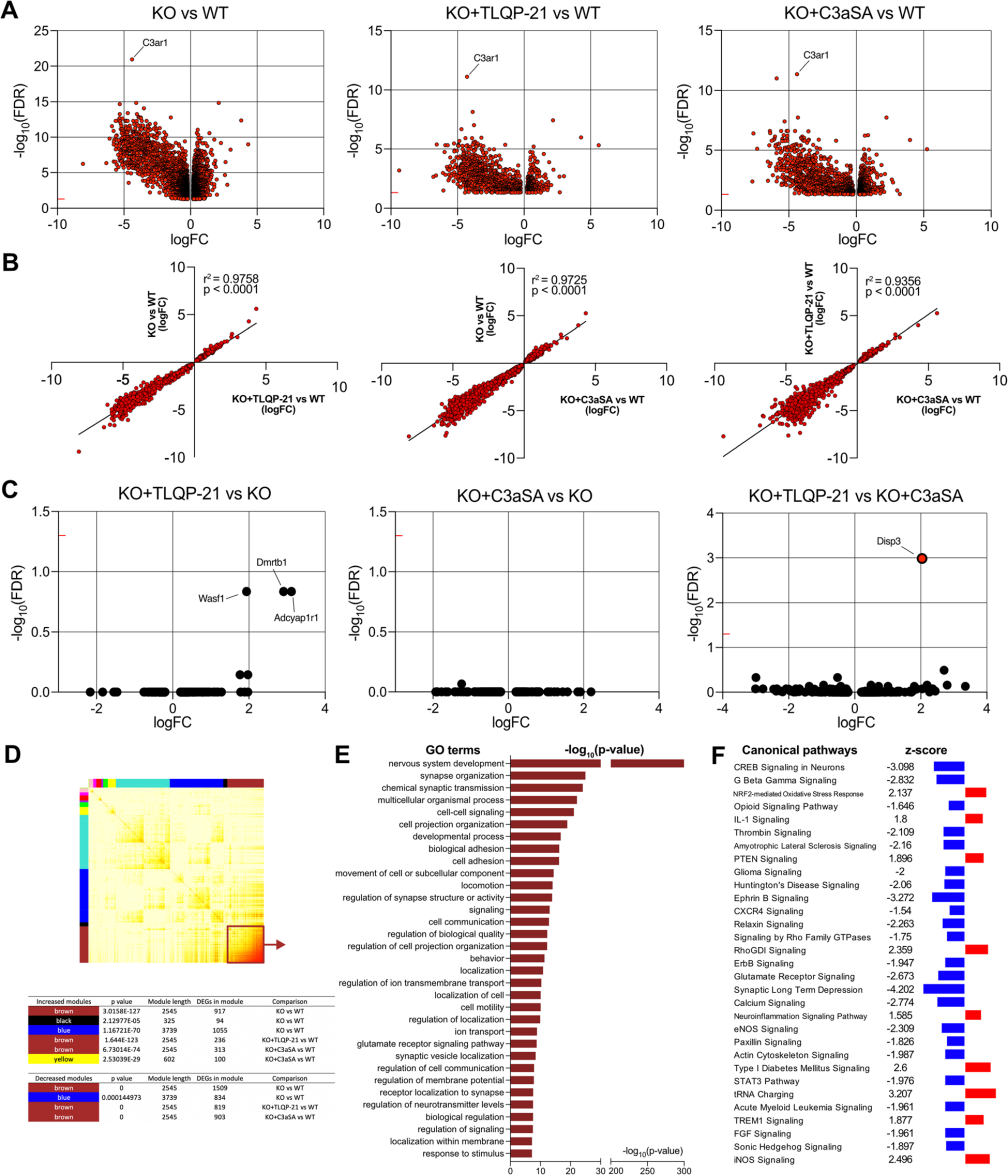
C3aR1 knock-out abolishes TLQP-21 induced DEGs. **a**, **b**, Volcano plot representations of the DEGs in WT or C3aR1 KO (KO) primary microglia, treated with vehicle, TLQP-21, or C3aSA. Only DEGs with FDR < 0.05 were represented (red dots). **c**, Correlation of fold changes in DEGs between KO vs WT and KO + TLQP-21 vs WT, KO vs WT and KO + C3aSA vs WT, KO + TLQP-21 vs WT and KO + C3aSA vs WT, respectively. **d**, Network modules identified by weighted gene co-expression network analysis (WGCNA). The co-expression network (20 samples in total) was constructed using all WT and KO-related comparisons. **e**, Sunburst representation of Gene Ontology (GO) analysis enriched in the “brown” module. **f**, Selected canonical pathways analysis (using Ingenuity Pathway Analysis (IPA) software) predicted to be increased (red) or decreased (blue) in the KO microglia compared to WT (full list in Additional file 2: Table S2)

When the transcriptomes of C3aR1 KO and WT primary microglia were compared, we identified 4943 DEGs, 2573 of which were downregulated and 2370 of which were upregulated (FDR < 0.05; − 0.2 < logFC<+ 0.2 were not considered; Fig. 5a; Additional file 1: Table S1). Comparing C3aR1 KO cells treated with TLQP-21 or C3aSA with WT (KO + TLQP-21 vs WT and KO + C3aSA vs WT respectively) resulted in a similar distribution pattern of the DEGs in the volcano plots (Fig. 5a). Correlation analyses between the comparisons KO vs WT compared to KO + TLQP-21 (r^2^ = 0.9758, *p* < 0.0001) or KO + C3aSA vs WT (r^2^ = 0.9725, *p* < 0.0001) and KO + TLQP-21 vs WT compared to KO + C3aSA vs WT (r^2^ = 0.9356, *p* < 0.0001) exhibited high correlation coefficients (Fig. 5b). Moreover, there were no DEGs in the KO + TLQP-21 vs KO or KO + C3aSA comparisons, consistent with the lack of effect of TLQP-21 or C3aSA on the transcriptome when C3aR1 is absent (Fig. 5c). Although these did not reach statistical significance, there was a strong upward trend in the KO + TLQP-21 vs KO comparison that are absent from the KO + C3aSA vs KO comparison in levels of *Wasf1* (logFC = 1.936739127; *p*-value = 2.50E-05; adjusted *p*-value = 0.146110951), *Dmrtb1* (logFC = 2.907497541; *p*-value = 2.45E-05; adjusted *p*-value = 0.146110951) and *Adcyap1r1* (logFC = 3.114420357; *p*-value = 3.40E-05; adjusted *p*-value = 0.146110951). It is possible that these 3 genes are upregulated after the interaction of TLQP-21 with another receptor that remains to be identified.

A co-expression network using all WT and KO comparisons, i.e. not restricted to the WT comparisons used in the Fig. 4 co-expression network, identified a co-expression module (“brown”) that was significantly enriched (FDR < 0.05) for DE genes up- or down-regulated for both KO vs WT and KO + TLQP-21 (or C3aSA) vs WT comparisons (Fig. 5d). Almost half of the DEGs identified in KO microglia compared to WT are present in this module, which is associated with several GO terms that suggest C3aR1 functions (Fig. 5e). Many of these overlap with the blue module described above, and include nervous system and synapse organizational terms and cell movement and migration. We also used Ingenuity Pathway Analysis (IPA) to identify canonical pathways predicted to be significantly (FDR < 0.05) increased (z-score > 2) or decreased (z-score < 2) in KO compared to WT (Fig. 5f, Additional file 2: Table S2), which included CREB, Calcium or Glutamate Receptor signaling, and importantly, as recently described, confirmed the predicted decrease of the STAT3 pathway in C3aR1 KO microglia [29].

### Chronic intracerebroventricular infusion of TLQP-21 reduces amyloid plaque load, microgliosis, and the number of dystrophic neurites in male 5xFAD mice

**Fig. 6:**
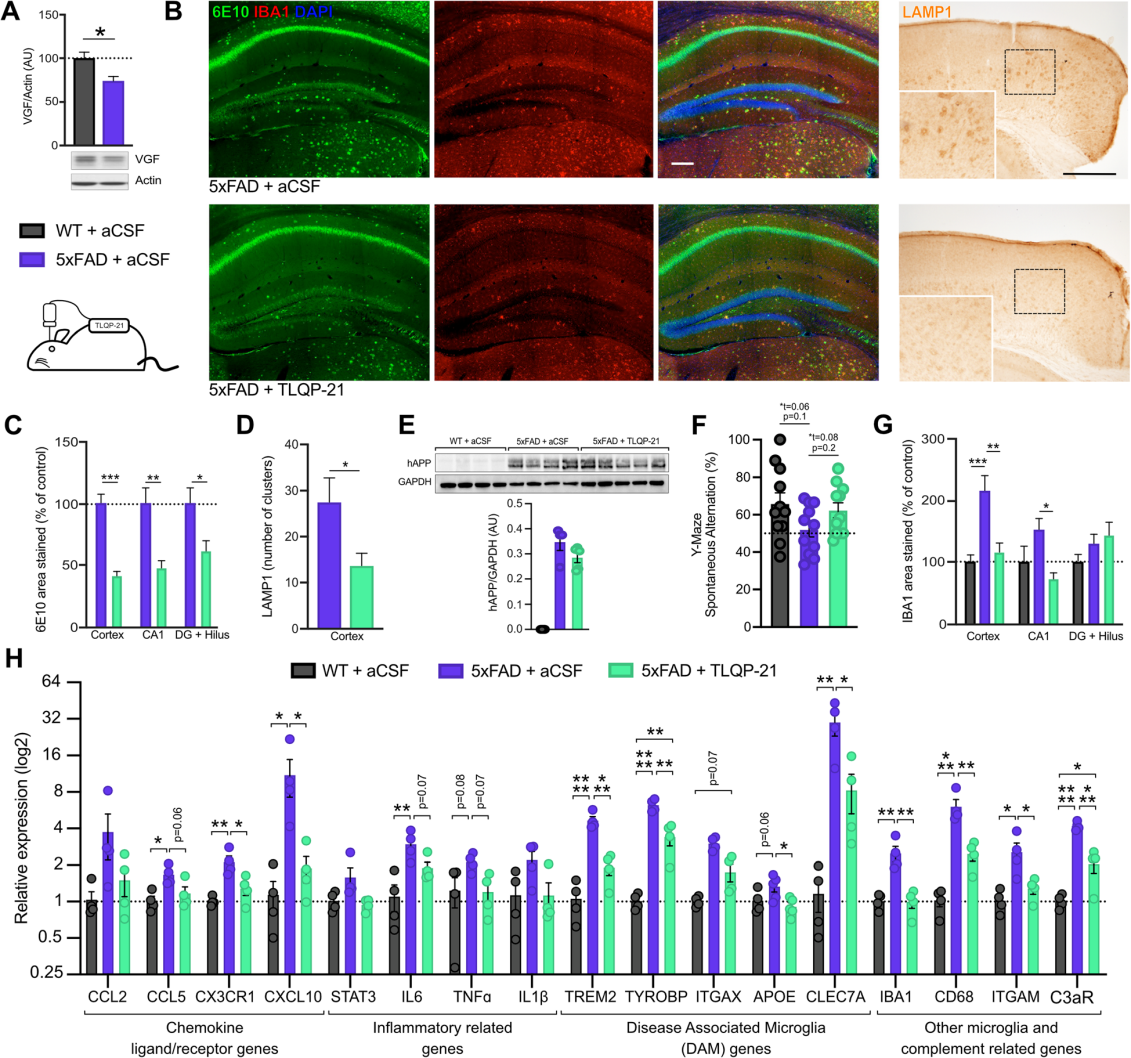
Chronic ICV administration of TLQP-21 reduces amyloid plaque load and restores expression of subsets of Alzheimer disease-associated microglial genes in 5xFAD male mice. **a**, Top: Western-blot and quantification of brain VGF protein expression in WT and 5xFAD mice, *n* = 4 male mice per group. Bottom: schematic representation of the intracerebroventricular infusion of TLQP-21 (or aCSF) performed on WT or 5xFAD mice at 3 months of age for 28 consecutive days. **b**, Left: Representative images of Aβ plaques (6E10, green) and surrounding microglia (IBA1, red) in hippocampi of 5xFAD male mice treated with TLQP-21 or aCSF. Scale bar = 200 μm. Right: Representative images of dystrophic neurites labelled with anti-LAMP1 (DAB immunohistochemistry) in the cortical areas of WT and 5xFAD mice treated with aCSF or TLQP-21. Scale bar = 500 μm. **c**, Quantification of the Aβ plaque area in brain regions (cerebral cortex, hippocampus CA1, and dentate gyrus + hilus), *n* = 4–5 male mice per group. 2 brain sections per animal were used for the analysis. **d**, Quantification of dystrophic neurite clusters illustrated in B, *n* = 5 male mice per group. 1 brain section per animal was used for analysis. **e**, Western blots from cortices of WT or 5xFAD male mice injected with aCSF and TLQP-21 using 6E10 antibody for human APP, *n* = 4–5 male mice per group. **f**, Percentage of spontaneous alternations between both arms of the maze, *n* = 11–12 male mice per group. **g**, Quantification of the area occupied by microglia in brain regions (cerebral cortex, hippocampus CA1, and dentate gyrus + hilus), *n* = 4–5 male mice per group. 1 brain section per animal was used for analysis. **h**, RT-qPCR analysis of microglial genes in the hippocampus of the 3 groups presented in (**e**), *n* = 4 male mice per group. Error bars represent means ± SEM. Statistical analyses were performed using a Student-t-test for A, C, D and F(*t). A One-Way ANOVA followed by a Tukey’s post-hoc test was used for G and H, *p < 0.05; **p < 0.01; ***p < 0.001, ****p < 0.0001

To evaluate the potential impact of TLQP-21 administration on amyloid phenotype in a mouse model, osmotic pumps were implanted in 3 month old male and female 5xFAD, a transgenic mouse amyloidosis model that expresses five familial AD mutation in APP and presenilin [34]. Notably, VGF protein level in dorsal hippocampus is significantly reduced in 5xFAD compared to wild-type littermate controls (Fig. 6a). Following TLQP-21 infusion for 28 days, mice were sacrificed at 4–5 months of age. As Aβ plaques are surrounded by activated microglia in AD [49, 50], we analyzed co-localization of plaques and microglia in 5xFAD mice treated with TLQP-21 or aCSF by co-staining with anti-Aβ (6E10) and anti-IBA1 antibodies. In male mice, infusion of TLQP-21 produced a 50% decrease of 6E10-immunoreactive plaques associated with fewer reactive microglia in cortex, CA1, and dentate gyrus and hilus (Fig. 6b-c). No differences in 6E10 staining were observed in brain regions from female 5xFAD treated with TLQP-21 or aCSF (Additional file 3: Figure S1), so all data shown below are from males. In addition to reactive glial cells, amyloid plaques are also surrounded by swollen presynaptic dystrophic neurites that consist of dysfunctional axons and terminals, and staining with anti-LAMP1 reveals the lysosomal vesicles enriched in these dystrophic neurites. Dystrophic neurite cluster numbers in 5xFAD cortex and hippocampus were reduced by ~ 50% following TLQP-21 treatment (Fig. 6b-d). Levels of human APP (Fig. 6e) and the post-synaptic protein PSD-95 (Additional file 4: Figure S2) were unchanged in 5xFAD mice at this age. Using the hippocampus-dependent Y-maze task to assess spatial memory, we observed a trend to a memory deficit in aCSF-treated 5xFAD mice (*n* = 11 mice per group; *p* = 0.1 by ANOVA—trend analyzed by Student’s t test comparing 5xFAD to WT, t = 0.06), and a small but not statistically significant rescue by TLQP-21 (trend, *p* = 0.2 by ANOVA—trend analyzed by Student’s t test comparing 5xFAD/aCSF to 5xFAD/TLQP-21, t = 0.08) (Fig. 6f). Associated with the decrease in plaques, there was a reduced microglial density in 5xFAD mice infused with TLQP-21 compared to aCSF (Fig. 6g). Restoration of Iba1 density by TLQP-21 infusion, comparable to WT levels, was associated with a significant reduction in the expression of many microglial genes whose expression is increased in the context of AD (Fig. 6h). Taken together, these results indicate that chronic icv TLQP-21 administration significantly reduces amyloid neuropathology and microgliosis in male 5xFAD mice, but somewhat surprisingly, had no effect in female 5xFAD mice.

### Human TLQP-21 (hTLQP-21) and C3aSA peptides similarly activate HMC3 microglia

**Fig. 7:**
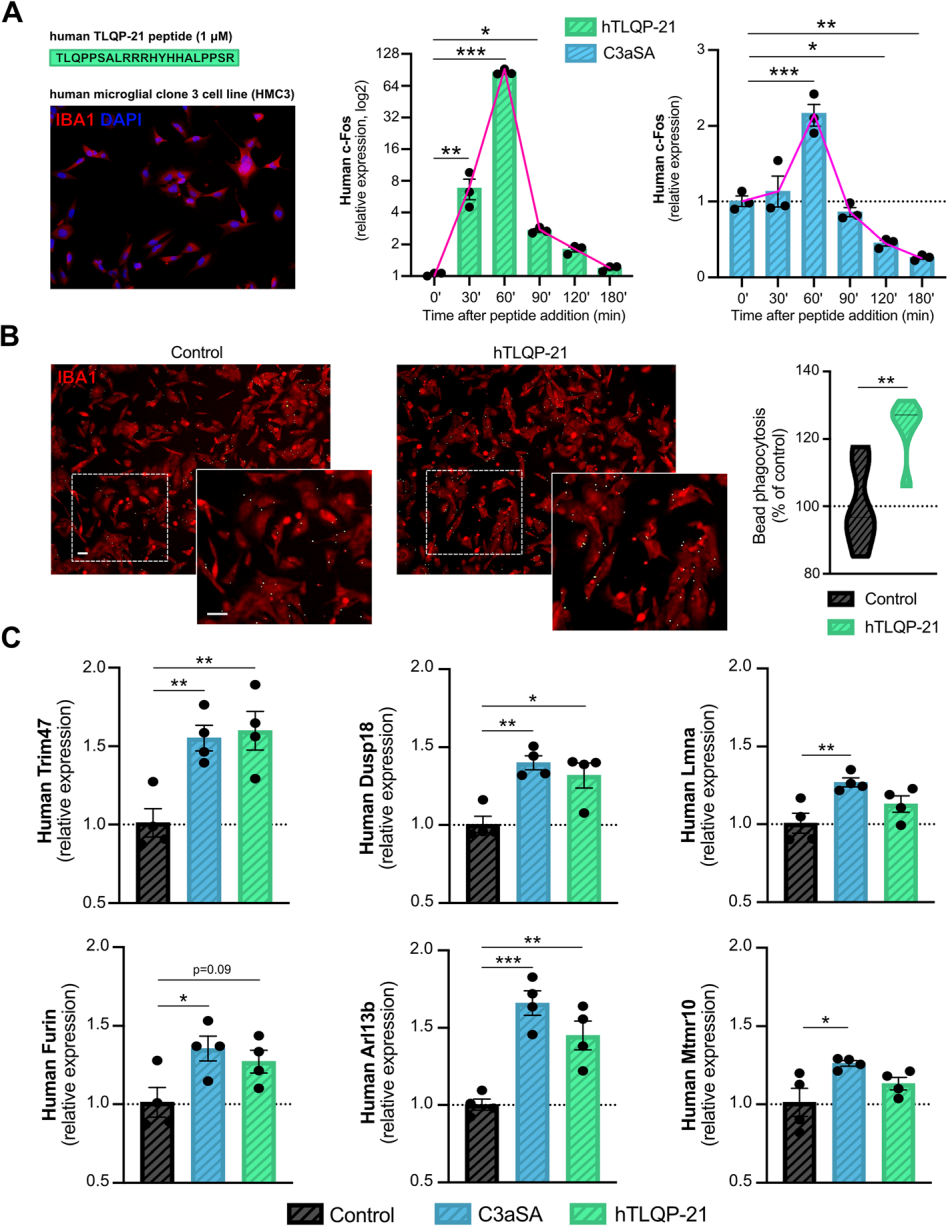
Human TLQP-21 and C3aSA similarly activate human microglia. **a**, Left: The sequence of the human TLQP-21 (hTLQP-21) peptide and a photomicrograph of the human microglial clone 3 cell line (HMC3) immunostained with the microglial marker anti-Iba1, are shown. Right: RT-qPCR quantification of human c-Fos expression after treatment (0 to 180 min) of HMC3 cells with 1 μM of hTLQP-21 or C3aSA, *n* = 3 per group. **b**, Representative images (Left) and quantification (Right) of latex bead phagocytosis assay on HMC3 microglia treated with or without 1 μM of hTLQP-21 for 1 h. Scale bar = 50 μm. **c**, RT-qPCR analysis for the human forms of the top 6 targets identified by the murine primary microglia RNA sequencing after treatment with the murine form of TLQP-21 or C3aSA (see Fig. 4) in HMC3 treated with or without hTLQP-21 or C3aSA for 24 h, *n* = 4 treated wells per group from 2 independent experiments A Kruskal-Wallis test was used for A, a Student-t-test for B and a One-Way ANOVA followed by a Tukey’s post-hoc for C, *p < 0.05; **p < 0.01; ***p < 0.001

As a proof of concept to evaluate the relevance of TLQP-21 in human, we treated the human microglial HMC3 cell line with hTLQP-21 peptide or C3aSA which produced a transient, robust increase in the expression of the immediate early gene *c-Fos* (Fig. 7a). Phagocytic function was quantified and similar to what was observed in murine BV2 and primary microglia, bead phagocytosis increased 25% following hTLQP-21 treatment (Fig. 7b). Finally, HMC3 cells were treated with hTLQP-21 or C3aSA and RT-qPCR assays were performed for the 6 most highly altered DEGs in murine primary microglia treated with TLQP-21 or C3aSA. *Trim47*, *Dusp18* and *Arl13b* were significantly increased by approximately 50%, and a trend was observed for *Lmna*, *Furin* and *Mtmr10* (Fig. 7c). Together, these data confirm the potential relevance of the mechanisms and pathways that we observed in murine cells and mice, for human disease.

## Discussion

VGF expression is reduced in the brains of patients with neurodegenerative disease, including AD, Parkinson’s disease, and amyotrophic lateral sclerosis (ALS) [51, 52]. Moreover, a number of biomarker studies have identified decreased VGF-derived peptides in the cerebrospinal fluid (CSF) from AD patients relative to controls [10–12, 14, 53, 54]. High-resolution proteomics identified VGF as a strong candidate biomarker of AD progression, with an estimated 10% decrease in CSF levels of VGF per year in diseased patients but not in age-matched controls [10]. A prospective study demonstrated reduced VGF levels in CSF from patients with mild cognitive impairment, but only in those who progress to clinical AD [12, 55]. VGF is robustly increased by exercise [56], which delays phenotype in AD mouse models [6]. It is also increased by environmental enrichment (EE) [57], which reduces amyloid toxicity and AD-like phenotypes and prevents microglial-mediated neuroinflammation [58–62]. We hypothesized that reduced VGF may be mechanistically involved in AD pathogenesis and/or progression [15], particularly via activity of TLQP-21. We first showed that TLQP-21 activates microglia via C3aR1. We then showed that VGF protein is reduced in the 5xFAD mouse, and that icv administration of TLQP-21 reduced its neuropathological phenotype and had a minor effect on modified Y-maze behavior in young mice, in which synaptic abnormalities are not yet detectable [34, 63]. Notably, these changes were associated with a restored transcriptome and microglial phenotype. Finally, by treating HMC3 microglia with hTLQP-21, we established that these observations generalize to human cells and potentially human disease. This particular, novel activity of TLQP-21, likely via C3aR1, may represent a new therapeutic approach for the treatment of AD. Recently described differences between human and mouse C3aR1 sequences and structure/function relationships in the context of TLQP-21- enhanced adrenergic receptor-induced lipolysis in adipose tissue [64] suggest future investigations of dose response relationships, downstream signaling, calcium mobilization, and transcriptional regulation will be required to fully determine hTLQP-21-receptor interactions critical for human microglial function.

To determine the potential mechanisms(s) via which TLQP-21 infusion reduced amyloid plaque load in 5xFAD, we investigated peptide actions in vitro, focusing on microglia. TLQP-21 induced a phenotypic alteration of BV2 microglia, which frequently appear as rod-shaped/elongated cells. This microglial phenotype is associated with increased cell motility/migration and phagocytosis, and also with a highly proliferative state that is observed at sites of CNS injury and repair [65–68]. During the early phase of brain injury, bipolar/rod-shaped microglia accumulate at the site of injury, which is crucial for minimizing further damage and facilitating repair, as reducing the number of proliferating microglia results in more severe damage to the cortex after ischemic insult [69, 70].

C3aR1 is a receptor for TLQP-21 [5, 24, 25, 29] and is predominantly expressed by microglia in the brain [71]. C3 is specifically released by astrocytes [71] and VGF is primarily produced by neurons, with a very low level of expression in microglia [72]. This suggests an essential role for microglial C3aR1 in the communication between neurons, astrocytes, and microglia. In another example of such communication between cell types, TLQP-21 has a dose-dependent pronociceptive effect in spinal cord through the activation of dorsal horn microglia in the spared nerve injury (SNI) model of neuropathic pain [47, 73]. VGF expression is induced in dorsal root ganglion (DRG) neurons within 24 h of injury and persists for at least 7 days, and VGF-derived peptides, including TLQP-21, may contribute to the development and maintenance of nerve injury induced hypersensitivity [73, 74]. In fact, the pronociceptive effect of TLQP-21 is mediated by C3aR1-expressing microglia in the spinal cord [47]. TLQP-21 evokes Ca^2+^ transients in microglia that is C3aR1-dependent and can be blocked by C3aR1 inhibitor or C3aR1 gene deletion, while both C3aR1 antagonist and TLQP-R21A reduced spinal nerve injury-induced hypersensitivity in the mouse SNI model [47].

The RNA sequencing provides further support for C3aR1 as the primary TLQP-21 receptor on microglia. Thus, TLQP-21 and C3aSA induced almost identical transcriptomic changes, with upregulation of most of the DEGs, many of which are associated with cellular movement and proliferation. They include *Arl13b*, which is involved in cell projection and primary cilia formation [75, 76]. *Dpysl2* encodes a member of the collapsing response mediator protein family (CRMP2) facilitating growth, guidance or polarity by having functions related to the cytoskeletal dynamics [77]. *Klf4* encodes a zinc-finger transcription factor also involved in the regulation of proliferation and differentiation [78–80]. *Cd44* encodes a cell-surface glycoprotein involved in cell-cell interactions, cell adhesion and migration [81]. *Lmna* encodes the A-type lamin, a major component of the nuclear lamina also involved in cellular proliferation and differentiation [82]. It is conceivable, therefore, that these gene products altered by TLQP-21 may participate in the changes in the microglia in 5xFAD mice after infusion. Interestingly, both CD44 and LMNA proteins have been recently described among a six protein module/hub (with PLEC, MSN, ANXA5 and GFAP) that are enriched in astrocytes/microglia and positively correlate with AD stages [83].

The *Trim47* and *Dusp18* genes exhibit the highest log (2)-fold changes following exposure of microglia to TLQP-21. *Trim47* encodes a member of the TRIM family of E3 ubiquitin ligases which regulate immune signaling pathways within microglia [84, 85] and is loosely associated with several neuropsychiatric diseases [86]. *Dusp18* encodes a dual-specificity phosphatase which inhibits the SAPK/JNK signal pathway [87]. An increase of DUSP18 may lead to a decreased activation of the SAPK/JNK pathway and thereby a reduction of *Jun* transcription, which is indeed the only downregulated DEG in microglia exposed to TLQP-21. Levels of phosphorylated JNKs are elevated in human post-mortem AD brain and JNK3 in particular enhances Aβ production and neurofibrillary tangle formation [88–91]. Consequently, JNK may be an attractive AD therapeutic target and several JNK inhibitors have been developed [92], but approaching this pathway via TLQP-21 may be another option. Interestingly, *Trim47* and *Dusp18* were increased in human microglia exposed to human TLQP-21 peptide. This provides an interesting proof of concept for human relevance, indicating that human and mouse microglia respond similarly to both human and mouse TLQP-21, respectively.

In a previous study, administration of the C3aR1 antagonist SB290157 to APPswe/PS1ΔE9 transgenic mice led to a decrease in Aβ pathology and microgliosis [71], contrasting with our finding that TLQP-21, acting as a C3aR1 agonist, also reduced microgliosis and amyloid load. Notably, 10 μM SB290157 acts as a C3aR1 agonist rather than as an antagonist [93, 94]. We also exposed BV2 cells to 0.25–1 μM of SB290157 and observed a significant increase of several immediate early genes by RT-qPCR, again implying agonist activity (data not shown). We therefore concluded that use of C3aR1 KO microglia is preferable to pharmacologic antagonism. In vivo, germline or conditional ablation of C3aR1 in APP/PS1 or 5xFAD amyloidosis mouse models has not been reported. Pathology in the PS19 tauopathy model is reduced when they are crossed with constitutively null *C3aR1* mice [29] but it is possible that *C3aR1* deletion may have significantly different effects on amyloidosis. Interestingly, as part of the tauopathy study, Zheng and colleagues detected only 38 DEGs comparing C3aR1 KO to WT adult unfractionated hippocampus (FDR < 0.1, 29], whereas there were 4943 DEGs in our comparison of C3aR1 KO and WT primary microglia cultured from P0 cerebral cortex (FDR < 0.05), with only 4 overlapped with the 38 and which moved in opposite directions (personal communication). Similarly, C3aR1 activation via TLQP-21 or C3aSA resulted in a low number of DEGs. These data may suggest that in vivo, complement production and activation of C3aR1 via C3a regulates homeostatic gene expression during development, limiting the changes in the microglial transcriptome. Lastly, a previous report indicates that short-term (1 h) and long-term (24 h) treatment with C3a increases and decreases, respectively, primary microglial phagocytosis [71], suggesting that prolonged and acute C3aR1 activation may differentially regulate microglial function. Therefore, the developmental timing of C3aR1 ablation and the length of time that the C3aR1 pathway is activated or inhibited may have critical effects on amyloid or tau neuropathology in mouse models.

It is intriguing that *Cd33* is upregulated in the KO vs WT and KO + C3aSA vs WT comparisons. CD33 is expressed in microglia and is increased in AD. CD33 reportedly inhibits microglial uptake of Aβ42 and its suppression stimulates phagocytosis and retards plaque growth [95]. It is possible, therefore, that increased CD33 may be directly associated with reduced phagocytosis, as seen in the *C3aR1* KO microglia (see Fig. 3). However, determining the precise role that CD33 plays in microglial function and phagocytosis is complex, and is complicated by species-specific differences between human and mouse CD33 in sialic acid-dependent binding (e.g. to alpha2–3- or alpha2–6- linked sialic acids by human but not mouse CD33, [96] and in intracellular CD33 signaling motifs [e.g. two immunotyrosine inhibitory motifs (ITIMs) present in human but not mouse CD33 [97, 98]. Moreover, predictions based on increased CD33 expression and increased plaque burden in human AD brains [99, 100] were not at first glance completely consistent with the outcomes of experiments that reduced CD33 expression in knockout mice and BV2 murine microglia, which resulted in impaired uptake and clearance of Aβ42 in cultured microglia and reduced plaque burden and insoluble Aβ42 in APP/PS1/CD33^−/−^ mice [95].

Lack of a significant decrease in amyloid plaque load in TLQP-21-treated 5xFAD females was surprising, despite an observed trend, but could reflect more aggressive amyloid deposition at younger ages in female compared to male 5xFAD mice [34], which was also observed in APP/PSEN1 mice [101]. Indeed, female mice express higher levels of APP than males, which generates higher levels of Aβ, possibly due to an estrogen response element in the Thy1 promoter that is used in the 5xFAD mouse model to drive transgene expression [34, 102, 103]. Considering that neuropathology in female 5xFAD is worse than in males at a given age, and that TLQP-21 infusion was limited in duration (28 days), it is conceivable that peptide administration for this duration at this age was insufficient to reverse or retard development of neuropathology in females as efficiently as in males. This phenotypic difference between male and female 5xFAD mice is also consistent with the higher incidence of AD in female compared to male human subjects. Interestingly, TLQP-21 is also known to be involved in the regulation of the reproductive cycle in female rodents [18, 21], and Broestl and colleagues have observed that female hAPP mice in estrogen-dominant cycle stages have worsened AD-related network dysfunction and cognitive impairments, while in contrast, those in progesterone-dominant stages and after gonadectomy have attenuated AD-related deficits [104]. Failure to detect equivalent effects of TLQP-21 treatment in male and female 5xFAD could also reflect sex-related differences in microglial function, which could be associated with transcriptional and translational differences between male and female microglia, including in C3aR1 expression levels which are higher in males [105, 106]. Thus, failure of TLQP-21 to reverse neuropathology in females could have complex underlying mechanisms that are based on estrogen or progesterone levels, and in our studies, we did not investigate actions of icv-infused TLQP-21 on the female 5xFAD reproductive cycle or circulating reproductive hormones. TLQP-21 is also known to induce acute gonadotropin responses in pubertal and adult male rats and to stimulate luteinizing hormone (LH) secretion in pubertal males [2], but icv TLQP-21 treatment of 5xFAD males still reduced neuropathology independent of any actions that it could have had on male reproduction or reproductive behavior, which we did not specifically assess in our studies.

Our studies of peptide-treated male and female 5xFAD mice shown here assessed delivery of a single dose of peptide, and peptide administration was initiated at a single age. Also, we analyzed results at a single time point following peptide administration. Further experimentation is required to determine whether the changes we observed in amyloid load, microgliosis, astrogliosis, and/or behavior would be further altered by changes in the age of the mice, either younger or older, the amount of administered peptide, and/or the length of time of peptide delivery if assessed in older or younger age mice. It is also possible that one or more of these protocol changes would alter the gender-specific efficacy of TLQP-21. The answers to these questions will be required to fully assess the potential pharmacotherapeutic utility of VGF-derived peptides to reverse or delay AD pathogenesis and progression.

Lastly, spontaneous alternation behavioral responses of 5 month-old 5xFAD male mice in the Y-maze task were determined immediately following completion of the 28-day icv infusion of TLQP-21. In the original description of the 5xFAD mouse line by Oakley et al. [34], ~ 5 month-old 5xFAD mice were found to have a Y-maze spatial memory deficit. However, in two subsequent studies, a significant memory deficit in the Y-maze was not detected at this age [44, 45]. We observed a trend to minor memory impairment in the aCSF-infused 5 month-old 5xFAD cohort that was partially rescued in TLQP-21-treated mice (trend) (Fig. 6f). These trends are consistent with the neuropathological rescue we observed at this age, and also with parallel studies that utilized long-term viral or transgenic VGF overexpression in hippocampus, so that older 5xFAD mice were assayed. In these latter experiments, Barnes maze memory in VGF-overexpressing 7 month-old 5xFAD was rescued, as were LTD deficits in hippocampal slices from 9 month-old VGF-overexpressing 5xFAD male mice [15] (Beckmann, Lin et al., 2019, submitted).

## Conclusions

Herein, we show that TLQP-21 activation of C3aR1 increases microglial migration and phagocytosis, and that icv TLQP-21 administration to male 5-month-old 5xFAD mice reduces amyloid plaque burden and microgliosis, and restores expression of a subset of AD-associated genes to wild type levels. Because reduced levels of protein markers of synaptic health (e.g. PSD-95) are generally not observed in 5xFAD until they are at least 6-months of age [34, 63, 107, 108], future experiments will be required to determine whether TLQP-21-mediated rescue of amyloid and microglial phenotypes in male 5xFAD mice at 5 months of age is associated with memory improvement and reduced synaptic damage as the TLQP-21-treated mice age. Importantly, we find that human TLQP-21 activates human microglia with similar outcomes to the activation of murine microglia by mouse TLQP-21, confirming the potential relevance of the mechanisms and pathways that we described for human disease. Work is in progress to determine whether C3aR1 is necessary for the entire beneficial effect of TLQP-21 and VGF in vivo and whether gene expression changes in TLQP-21-exposed microglia may lead to novel therapeutic approaches.

## Supplementary information

**Additional file 1: Table S1.** List of Differential Expressed Genes (DEGs) for the following comparisons: C3aR1 vs WT, C3aR1+TLQP-21 vs WT and C3aR1+C3aSA vs WT. DEGs have been sorted depending on whether they are down- or up-regulated.

**Additional file 2: Table S2.** Canonical Pathways and other analyses generated by the Ingenuity Pathway Analysis (IPA) software for the C3aR1 vs WT comparison.

**Additional file 3: Figure S1.** Area stained with 6E10 is decreased in 5xFAD males infused with TLQP-21. Comparison of total amyloid plaque area stained with anti-6E10 antibody in the brains of male and female 5xFAD mice infused with TLQP-21 or aCSF (control). Animal numbers used for the analysis: male, *n* = 4–5 per group; female, *n* = 4–7 per group. 1–2 brain sections per animal were used for the analysis. Error bars represent means ± SEM. Student t-test, *p < 0.05; **p < 0.01; ***p < 0.001. Abbreviations: cerebral cortex (Cortex), hippocampal CA1 (CA1), CA3 (CA3), and dentate gyrus+hilus (DG + Hilus).

**Additional file 4: Figure S2.** PSD-95 is unaltered in 5 months-old 5xFAD mice. Western blot and densitometric analysis from cerebral cortices of WT and 5xFAD mice infused icv with aCSF or TLQP-21 using anti-PSD-95 and anti-GAPDH antibodies, *n* = 4–5 male mice per group.

## Data Availability

All the primary data supporting the conclusions of this study are included in the manuscript and Supplementary tables, and are being deposited in SYNAPSE.

## Abbreviations

aCSF: Artificial Cerebrospinal Fluid
AD: Alzheimer’s Disease
AMP-AD: NIH Accelerating Medicines Partnership for Alzheimer’s Disease
C3aR1: Complement C3a receptor-1
C3aSA: C3a super agonist
CDR: Clinical Dementia Rating
CNS: Central Nervous System
DEG: Differentially Expressed Genes
DRG: Dorsal Root Ganglion
FDR: False Discovery Rate
ICV: Intracerebroventricular
IPA: Ingenuity Pathway Analysis
KO: Knock-Out
PNS: Peripheral Nervous System
WGCNA: Weighted Gene Co-expression Network Analysis
WT: Wild-Type
