# Supplementary figures and images for: VGF-derived peptide TLQP-21 modulates microglial function through C3aR1 signaling pathways and reduces neuropathology in 5xFAD mice
Source: Mol Neurodegener. 2020 Jan 10;15:4. doi: 10.1186/s13024-020-0357-x (PMC6954537; doi:10.1186/s13024-020-0357-x)

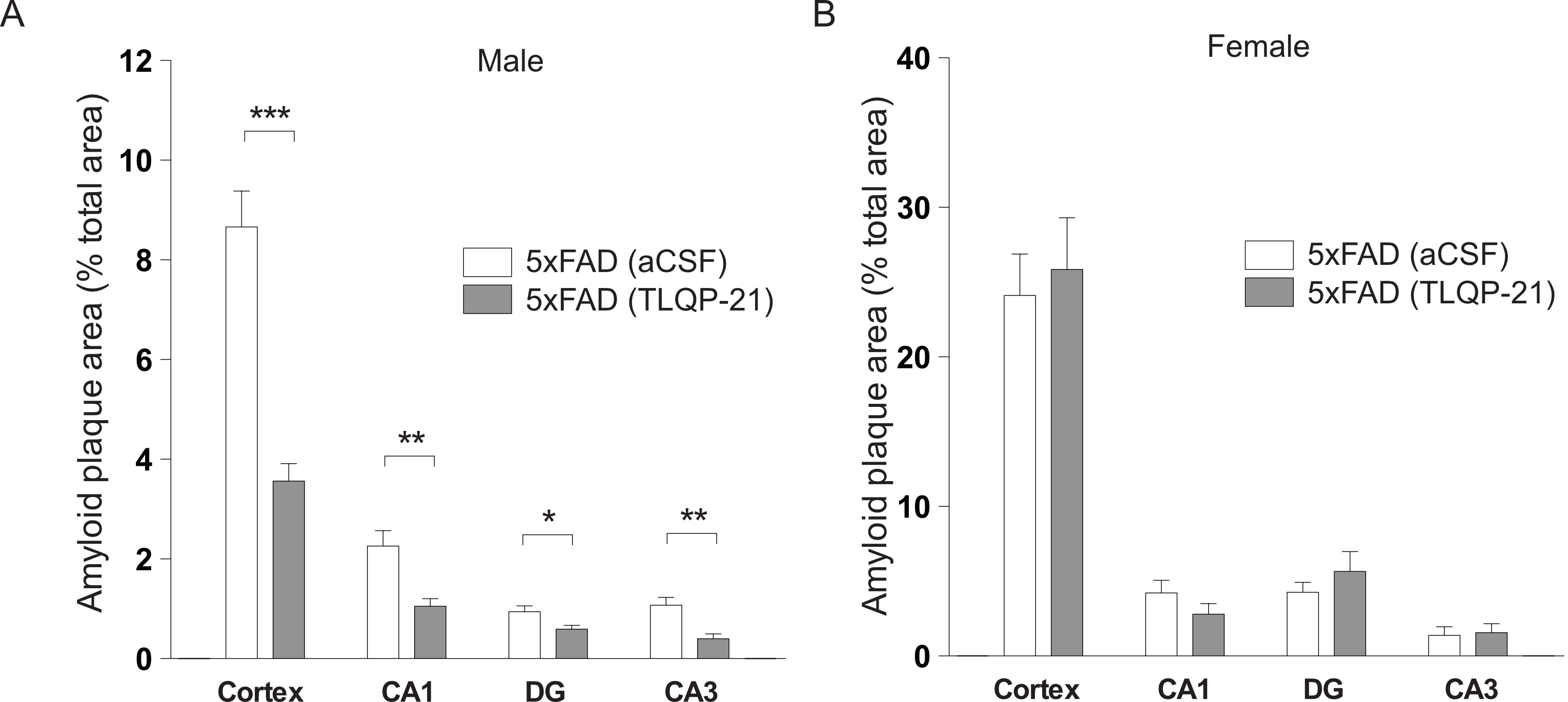

Supplement: Supplementary file 3 — Additional file 3: Figure S1. Area stained with 6E10 is decreased in 5xFAD males infused with TLQP-21. Comparison of total amyloid plaque area stained with anti-6E10 antibody in the brains of male and female 5xFAD mice infused with TLQP-21 or aCSF (control). Animal numbers used for the analysis: male, n = 4–5 per group; female, n = 4–7 per group. 1–2 brain sections per animal were used for the analysis. Error bars represent means ± SEM. Student t-test, *p < 0.05; **p < 0.01; ***p < 0.001. Abbreviations: cerebral cortex (Cortex), hippocampal CA1 (CA1), CA3 (CA3), and dentate gyrus+hilus (DG + Hilus). [file 13024_2020_357_MOESM3_ESM.tiff]

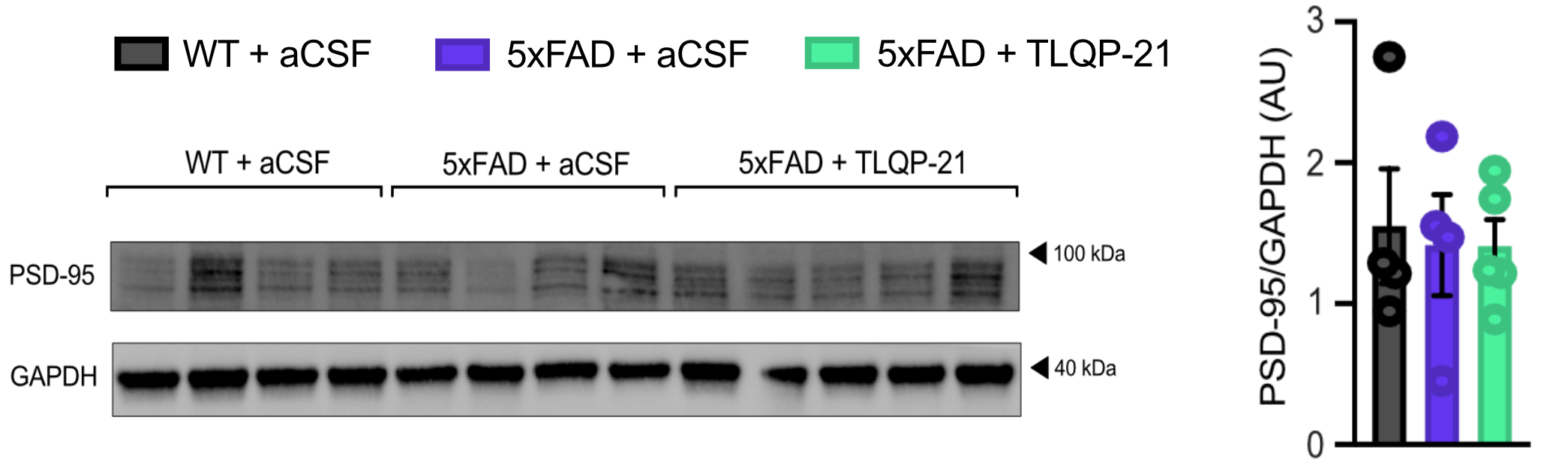

Supplement: Supplementary file 4 — Additional file 4: Figure S2. PSD-95 is unaltered in 5 months-old 5xFAD mice. Western blot and densitometric analysis from cerebral cortices of WT and 5xFAD mice infused icv with aCSF or TLQP-21 using anti-PSD-95 and anti-GAPDH antibodies, n = 4–5 male mice per group. [file 13024_2020_357_MOESM4_ESM.tiff]
